# Psychometric properties of the High Five Inventory in university students in Ecuador

**DOI:** 10.3389/fpsyg.2025.1490889

**Published:** 2025-04-02

**Authors:** Jessica V. Quito-Calle, Alejandro César Cosentino, Dalila M. González-González, Luis F. Guerrero-Vásquez

**Affiliations:** ^1^Psychology Research Group (GIPSI-SIB), Faculty of Psychology, Universidad Politécnica Salesiana, Cuenca, Ecuador; ^2^Department of Psychology, Faculty of Social Sciences, Universidad de Palermo, Buenos Aires, Argentina; ^3^Applied Embedded Hardware Research Group (GIHEA), Universidad Politécnica Salesiana, Cuenca, Ecuador

**Keywords:** factorial analysis, reliability, positive personality factors, validity, High Five Inventory

## Abstract

**Introduction:**

The High Five Inventory (HFI) was developed to assess five positive personality traits: erudition, peace, joviality, honesty, and tenacity. Research on positive personality dimensions remains limited, emphasizing the importance of validating assessment tools in different cultural contexts. This study aimed to analyze the psychometric properties of the High Five Inventory (HFI) in a sample of Ecuadorian university students.

**Methods:**

An instrumental study was conducted to evaluate the construct validity and reliability of the HFI. A confirmatory factor analysis (CFA) was performed to assess the inventory's factorial structure. The sample consisted of 1,007 students (403 women, 39.9%) from various faculties at Salesiana Polytechnic University in Ecuador. Prior to data collection, a linguistic review was carried out by a panel of experts, including faculty members, researchers, and students, to ensure clarity and comprehension of the items. The final version of the inventory was administered after obtaining informed consent.

**Results:**

The CFA results indicated an adequate model fit: χ^2^ (220) = 859.969, *p* < 0.001, robust CFI = 0.989, RMSEA = 0.054 (90% CI: 0.050–0.058), and SRMR = 0.045. Additionally, internal consistency was confirmed with Cronbach's Alpha (α) and McDonald's Omega (ω) coefficients ≥ 0.80, demonstrating strong reliability.

**Discussion:**

The findings confirm that the High Five Inventory–Ecuador (HFI-E) exhibits robust psychometric properties, supporting its use in assessing positive personality traits within the Ecuadorian population. The study highlights the relevance of validating personality assessments across different cultural contexts and underscores the potential applications of the HFI-E in psychological and educational settings.

## 1 Introduction

The study of personality is a topic that has been investigated since ancient times (Millon, 2012). Understanding personality is essential, not only for explaining human developmental processes but also for providing a comprehensive framework to interpret individuals' actions, emotions, and motivations (Roberts and Yoon, 2021). Studying its development is crucial as it allows for an understanding of behavior at both the individual level and within the broader societal context. Personality theory is notable for its relevance in studying human nature and serves as the foundation for understanding any discipline (Hogan and Sherman, 2020b). Predominant theories addressing this construct include psychodynamic theory, trait theory, and interpersonal theory. Psychodynamic theory, closely linked with clinical psychology, posits that childhood experiences are determinant in shaping adult personality. Trait theory, on the other hand, focuses on the classification and measurement of individual differences, providing a framework to understand human diversity through stable personality dimensions. Finally, interpersonal theory examines the role of social interaction in personality, exploring how relationships and group dynamics affect and influence subsequent interactions (Hogan and Sherman, 2020a).

In this theoretical framework, personality psychology has contributed to studying its characteristic traits. However, authors such as DeYoung (2010) acknowledge certain limitations that reflect research on biological foundations. In this context, his work represents a significant advancement by exploring how personality neuroscience is expanding the understanding of the biological systems underlying the Big Five traits: extraversion, neuroticism, agreeableness, conscientiousness, and openness to experience. Furthermore, Sheng-lan (2012) emphasizes the importance of personality research in the psychological domain, noting that positive personality theory focuses on qualities, positive psychological virtues, and aspects or processes contributing to the optimal functioning of individuals, groups, and institutions, highlighting the significance of positive dimensions in personality.

In this context, our article aims to delve into the psychometric properties of the High Five Inventory (HFI) (Cosentino and Castro Solano, 2017) within the Ecuadorian context. This area has received little attention to date, despite the model demonstrating superior properties compared to conventional models (Quito-Calle and Cosentino, 2024). Given that integrating these psychometric properties could enhance the understanding of personality, we propose an experimental protocol with Ecuadorian university students to validate the HFI and describe its psychometric properties.

The relevance of this test motivates its adaptation to the Ecuadorian context, bearing in mind that psychological tests developed in a particular cultural setting may not be suitable for another culture. Differences in values, norms, customs, and language can affect how individuals interpret and respond to test items. Adaptation ensures that the questions are culturally relevant and comprehensible to the target population. Adapting psychological tests to specific countries is crucial for ensuring the accuracy, fairness, and usefulness of psychological assessments, thereby facilitating a better understanding and support for individuals within their unique cultural contexts.

This article is organized as follows: Section 2 provides a detailed description of the development of the HFI for measuring positive personality traits, incorporating statistical and syntactical criteria through a comprehensive explanation of personality, the High Five Model, and its factors. Section 3 examines related work on the ICA and results derived from these investigations and adaptations. Section 4 highlights the significance of our research within the Ecuadorian population, emphasizing its relevance despite the limited existing research, and proposes the hypothesis that the High Five Inventory supports the quality and validity of the construct. Section 5 details the study participants, the instrument applied, and the procedure used to test the hypothesis. Section 6 presents findings obtained through confirmatory factor analysis and assesses the instrument's reliability. Finally, Section 7 discusses the results in relation to other empirical studies in the field, while Section 8 concludes with a summary of the study's objectives and overall findings.

## 2 Theorical background

Personality is conceptualized as a dynamic structure that determines behavior, thought, and individuals' ability to adapt to their environment (Simkin et al., 2012). In this context, the Five Factor Model (FFM), developed by Costa and McCrae (Costa and McCrae, 1992), emerges as an essential theoretical framework for understanding the basic dimensions underlying personality, making it one of the most studied and researched topics (Gosling et al., 2003).

Costa and McCrae (1992) emphasize that the five personality factors (neuroticism, extraversion, openness, agreeableness, and conscientiousness) are characterized by manifesting in four fundamental aspects. First, these factors can be expressed through observable patterns of behavior. Second, traits are present across various personality systems and are reflected in natural language. Third, these traits are evident in diverse variables such as age, gender, race, language, and culture. Finally, these traits have a biological basis. In addition, these factors allow for the analysis of personality disorder scales or related issues. Furthermore, Smith and Williams (1992) highlight the significant role of personality in modulating health, emphasizing how individual characteristics can influence physiological and emotional aspects of health. Research in this domain suggests that personality traits not only affect how individuals respond to physical and psychological stressors, altering the frequency, duration, and intensity of physiological responses but also influence susceptibility to experiencing negative or dysphoric emotional states (Carver and Connor-smith, 2010).

In the context of the Big Five personality Model (BFM), Whiteside and Lynam (2001) research sheds light on the fundamental role of impulsivity within the personality structure. This study highlights that impulsivity is not only crucial for understanding the intrinsic dynamics of personality but is also essential in diagnosing and comprehending various forms of psychopathology. The significance of this trait has been acknowledged to the extent that it has been included in the Diagnostic and Statistical Manual of Mental Disorders (DSM-5-TR), where diagnostic criteria directly related to impulsivity are specified. In contrast, Chavira Trujillo and Celis de la Rosa (2021) emphasize the relevance of applying the BFM to assess positive personality traits, particularly in culturally specific contexts such as Latin America. This approach suggests a complementary perspective on personality study, where identifying and enhancing positive factors can offer new avenues for personal development and psychological wellbeing. The diversity in the application and interpretation of these models reflects the complexity of human personality and the need to adapt assessment and understanding tools to varied cultural and clinical contexts.

In response, Castro Solano and Cosentino (2017) developed the HFM, which serves to identify positive human characteristics from the perspective of ordinary people. The HFM is presented as an applicable model of five positive personality factors: erudition, peace, joviality, honesty, and tenacity, known as high factors, which are conceptually and empirically proximate to the factors of the BFM: extraversion, agreeableness, conscientiousness, neuroticism, and openness to experience. However, despite the relationship between the two models, the high factors of the HFM are considered as positive poles of the Big Five factors, but they are not mere duplications or repetitions of the BFM factors. Thus, the high factor Erudition is positively associated with the factor Openness to Experience; the high factor Peace is associated with Emotional Stability (as opposed to Neuroticism); the high factor Joviality is linked to Extraversion; the high factor Honesty is associated with Agreeableness; and the high factor Tenacity is related to Conscientiousness. Therefore, according to Solano and Cosentino (2019), the HFM factors are relatively stable within each individual and are represented by positive psychological characteristics. In addition, they possess certain notable attributes: (a) they can be measured, (b) they vary between individuals, and (c) they may increase or decrease due to internal or external influences. The term “high” for the HFM factors was chosen to indicate that these factors are linked to characteristics valued highly or positively by ordinary people.

The HFI, based on the HFM, was adapted in Argentina by Castro Solano and Cosentino (2017) to measure the HFM factors, known as high factors: erudition, peace, joviality, honesty, and tenacity. This instrument was developed through a deeply psycholexic inductive procedure that started from the perspective of ordinary people on positive characteristics (moral or non-moral). They generated a list of 854 items that constituted the initial corpus of words. The corpus was comprised of words used by ordinary people in their daily lives. No limits were established on the types of positive characteristics that could be mentioned, so the corpus included moral traits (e.g., reliable), traits related to abilities (e.g., intelligence), and traits lacking moral connotations (e.g., serenity). Thus, the HFM, by adopting statistical and syntactic criteria rather than semantic selection criteria, excludes any theoretical influence from an academic standpoint, aiming to achieve replication across different populations (Solano and Cosentino, 2019).

As explained, the factors of the HFM are distributed across five sub-scales of socially shared positive human characteristics, which are detailed further in Table 1.

**Table 1:** Description of factors in the High Five Model.

**HFM factors**	**Characteristics**
Erudition	The positive trait of knowledge, focused on thinking of solutions, creating things, and having a desire to learn. It is expressed in positive characteristics such as being intelligent, wise, visionary, knowledgeable, ingenious, and clever.
Peace	It is the positive trait of balance, focused on thinking calmly, believing that there is a solution for everything, or that things happen in their own time. It is expressed in positive characteristics such as being patient, tolerant, calm, and serene.
Joviality	It is the positive trait of emotion, focused on the desire to make people laugh, have fun, and help others to have fun or come up with amusing ideas. It is expressed in positive characteristics such as humor, amiability, being funny, and entertaining.
Honesty	It is the positive moral trait, focused on the desire to show one's true self, speak the truth, or be a good person. It is expressed in positive characteristics such as loyalty, reliability, having values, transparency, and truthfulness.
Tenacity	It is the positive trait of willpower, focused on thinking about goals, achieving them, or believing that goals require effort. It is expressed in positive characteristics such as dedication, perseverance, effort, and industriousness.

At the same time, Castro and Cosentino's studies Solano and Cosentino (2019) on individual positive characteristics have found that the HFM is related to indicators of illness, the risk of non-communicable medical diseases, psychopathological symptoms, and psychopathological personality traits. In addition, it is associated with positive mental health and emotional, psychological, and social wellbeing from both hedonic and eudaimonic perspectives, and finally, with academic performance among university students.

## 3 Related works on High Five Inventory

In the field of HFM research, scientific production remains limited due to the recent introduction of this theoretical model. However, the studies conducted to date have yielded promising results, identifying consistent associations that favor the development of instruments to measure positive personality traits. Cosentino and Castro Solano (2017) in their study explored the additional external validity of the High Five Model, through the application of the High Five Inventory, which assesses five positive personality factors: erudition, peace, joviality, honesty, and tenacity. This instrument consists of 23 items with Likert-type scales ranging from 1 (never) to 7 (always) and was administered to 1,032 participants (512 men, 49.6%, and 519 women, 50.3%), with an average age of 39.42 years (SD = 14.33), residing in the Autonomous City of Buenos Aires (*n* = 702, 68%) and in the Conurbano Bonaerense (*n* = 330, 32%). Among the main findings, a good fit was observed in both the generation sample (e.g., CFI = 0.968) and the confirmation sample (e.g., CFI = 0.963). Finally, the alpha and omega reliability for each factor was above 0.80, which supports the internal consistency of the instrument.

Following the same line of research, Solano and Cosentino (2019) conducted a factorial analysis aimed at optimizing the instrument by reducing the number of items while preserving its psychometric robustness. The objective of their study was to develop a factorial model with at least four items per factor, ensuring an omega reliability coefficient ≥0.80 and achieving a strong data fit. The sample used for model exploration included 516 participants (286 women, M = 35.2, SD = 13.5, range 18–80 years), who evaluated the extent to which the items reflected their characteristics on a scale from 1 to 7. To identify the instrument's underlying structure, an exploratory factor analysis (EFA) was conducted, leading to the selection of a preliminary set of 57 items, organized into five factors associated with positive personality traits.

Subsequently, the authors reported that the sample used for model refinement consisted of 484 participants (285 women, M = 35.1, SD = 14.0, range 18–79 years), who once again evaluated the extent to which positive characteristics described them on the same 1 to 7 scale. To optimize the factorial structure and reduce the number of items, a new exploratory factor analysis (EFA) was conducted, ensuring that each factor retained at least four items with an omega reliability coefficient ≥0.80 and an adequate fit to the data. As a result, the Inventario de los Cinco Altos (ICA) was finalized with 23 items distributed across five sub-scales, representing socially shared positive human traits. Psychometric analyses, conducted using a robust diagonally weighted least squares estimator, indicated an optimal model fit (CFI > 0.95, SRMR < 0.05, RMSEA < 0.06), with omega coefficients exceeding 0.80 for each factor, supporting internal consistency. To confirm model stability, an additional sample of 1,118 participants (564 women, M = 40.4, SD = 14.2, range 18–92 years) was analyzed, once again verifying an adequate fit through robust analyses (CFI > 0.96, SRMR < 0.05, RMSEA < 0.07) and high reliability levels across all sub-scales (omega > 0.80).

Thus, the Inventario de los Cinco Altos (ICA) was established as a psychometrically robust measure, comprising 23 items distributed across five subscales representing socially shared positive human traits, with responses rated on a Likert scale ranging from 1 (never) to 7 (always).

## 4 Present study

The importance of expanding information and knowledge about the HFM, as well as the quality of validity and reliability of the HFI in its previous adaptations, underscores the necessity of employing it within the Ecuadorian population to assess positive personality factors, given its absence in this country. Therefore, we propose the following hypothesis:

Hypothesis 1: The High Five Inventory adapted for the Ecuadorian population is reliable and valid.

This hypothesis is proposed to demonstrate that the model achieves an adequate fit to the data, with values close to or exceeding a “robust” CFI = 0.95 and internal consistency with α≥0.80 for each factor or dimension, as demonstrated in its previous adaptations: HFI by Castro Solano and Cosentino (2017) and Solano and Cosentino (2019).

## 5 Materials and methods

### 5.1 Participants

A total of 1, 007 participants (*n* = 403 women, 39.9%) took part, with an average age of 21.88 years (*SD* = 3.69). When categorized by age, the majority fall within the 18 to 20 years of range (42.1%), with an age span of 27 years. Participants are from various faculties of the private university in Ecuador, including Social Sciences and Humanities (*n* = 285, 28.3%), Science and Technology (*n* = 399, 39.6%), Administration and Economics (*n* = 158, 15.7%), Life Sciences (*n* = 113, 11.2%), and Education (*n* = 52, 5.2%). This is a non-probabilistic convenience sample, with voluntary, consented, and anonymous participation. No financial compensation was provided for participation.

### 5.2 Instrument

#### 5.2.1 High Five Inventory

In 2017, Castro Solano and Cosentino (2017) developed a measurement instrument for the factors of the HFM: erudition, peace, joviality, honesty, and tenacity. The pencil-and-paper instrument consists of 23 items with scales ranging from 1 (never) to 7 (always). Higher scores on each sub-scale indicate a higher level of the corresponding positive factor. The HFI demonstrated a good fit to the data both for the initial sample [e.g., Comparative Fit Index (CFI) = 0.968] and for the confirmation samples (e.g., CFI = 0.963). The alpha and omega reliabilities for each factor were above 0.80.

#### 5.2.2 Procedure

Initially, a linguistic review of the instrument was conducted in two stages. In the first stage, a panel of university faculty researchers reviewed the measurement instruments, and their feedback was considered before presenting them to students for the pilot study. No items or factors were removed, rendering an additional content validation process unnecessary. In the second stage, the tests were administered to a group of 10 students who independently evaluated the linguistic comprehension of the instruments. As a result, Table 2 presents the finalized items, adjusted for the linguistic and cultural adaptation of the Ecuadorian population, represented as the Ecuadorian version of the High Five Inventory (HFI-E).

**Table 2:** Linguistic comprehension of the items in the high HFI-E.

**Items from the original version Cosentino and Castro Solano in 2017**	**Items with linguistic understanding**
I am intelligent	I am intelligent
I have humor	**I have a sense of humor**
I have wisdom	I have wisdom
I have loyalty	I have loyalty
I am nice	I am nice
I am fun	I am fun
I have dedication	I have dedication
I am trustworthy	I am trustworthy
I am visionary	I am a visionary
I am tolerant	I am tolerant
I am cultured	I am cultured
I have peace of mind	I have peace of mind
I am persistent	I am persistent
I am funny	I am funny
I have values	I have values
I have genius	**I have talent**
I have serenity	I have serenity
I am transparent	I am transparent
I have effort	I have effort
I have wit	**I am ingenious**
I am industrious	**I am determined**
I am true	I am true
I have patience	I have patience

Items in bold are those that were linguistically adapted for Ecuadorian context. Content of original test items was modified in the adapted version to better suit Ecuadorian population.

Once the linguistic revisions of the instruments were completed, the application process commenced. Consequently, the link containing the instruments was published at: https://ee.humanitarianresponse.info/x/ieKH1SuM, accompanied by an invitation to complete it within the virtual classrooms of the Cooperative Virtual Learning Environments at the higher education institution. Students from various programs voluntarily responded individually to the instruments through the provided link. Each participant was also able to view an informed consent form containing: study information, and a request for anonymous and voluntary authorization to participate in the research. The application took approximately 5 min due to the number of items to be answered in the psychological test.

#### 5.2.3 Data analysis

Once data were collected through the administration of the measurement instrument, validity and reliability of the inventory were assessed using confirmatory factor analysis (CFA) (Brown et al., 2012). This analysis employed the diagonal weighted least squares (DWLS) estimator (Du and Bentler, 2022), based on the polychoric correlation matrix, due to its capacity to handle ordinal variables and mitigate potential biases in the estimation of factorial parameters.

Subsequently, CFA was performed and refined using R-Studio (Hanke and Halchenko, 2011), specifically with the Lavaan package (Rosseel, 2012), employing the diagonal weighted least squares (DWLS) estimator. Expected fit indices included a χ^2^ test with significance ≥0.05, Robust Comparative Fit Index (CFI) ≥0.95, standardized root mean square residual (SRMR) ≤ 0.08, and root mean square error of approximation (RMSEA) ≤ 0.07 [90% CI 0.00 to 0.08]. This reflects a reasonable model fit to the data. In addition, structural relationships identified in CFA were graphically represented using Onyx (von Oertzen et al., 2015), a specialized software for structural model visualization. As a complement to CFA, a reliability analysis was conducted using Cronbach's Alpha α and McDonald's Omega ω coefficients (Revelle and Zinbarg, 2009), ensuring internal consistency across all sub-scales. Given that specialized literature recommends a sample size of at least 10 times the number of observed variables for CFA (Alavi et al., 2020), the analyzed sample (*n* = 1, 007) was deemed sufficient to ensure model stability and robustness. In addition, it exceeded the data threshold defining large samples in psychometric studies (Lakens, 2021).

## 6 Results

### 6.1 Validity and reliability of Ecuadorian version of the High Five Inventory

#### 6.1.1 Construct validity of the HFI-E through confirmatory factor analysis

A confirmatory factor analysis was conducted using adult data through the lavaan package in R software on the scores from the HFI to determine the validity of a factorial structure of a theoretical model that posits positive personality traits are characterized by a set of factors. These positive personality traits, or high factors of the FHM, are relatively stable within each individual and are represented by positive psychological characteristics. The model development replicated five positive factors from the HFM, namely erudition, peace, joviality, honesty, and tenacity. To assess the latent structure of the FHM factors, multivariate normality tests and confirmatory factor analyses were conducted. Consequently, the assumption of multivariate normality of the sample distribution was not verified using Mardia's test. Instead, a robust model was employed using the lavaan package in R. Three indices were used to evaluate the model fit to the data: Comparative Fit Index (CFI) (Bentler, 1990), root mean square error of approximation (RMSEA) (Tennant and Pallant, 2012), and standardized root mean square residual(SRMR) (Pavlov et al., 2021).

**Figure 1:**
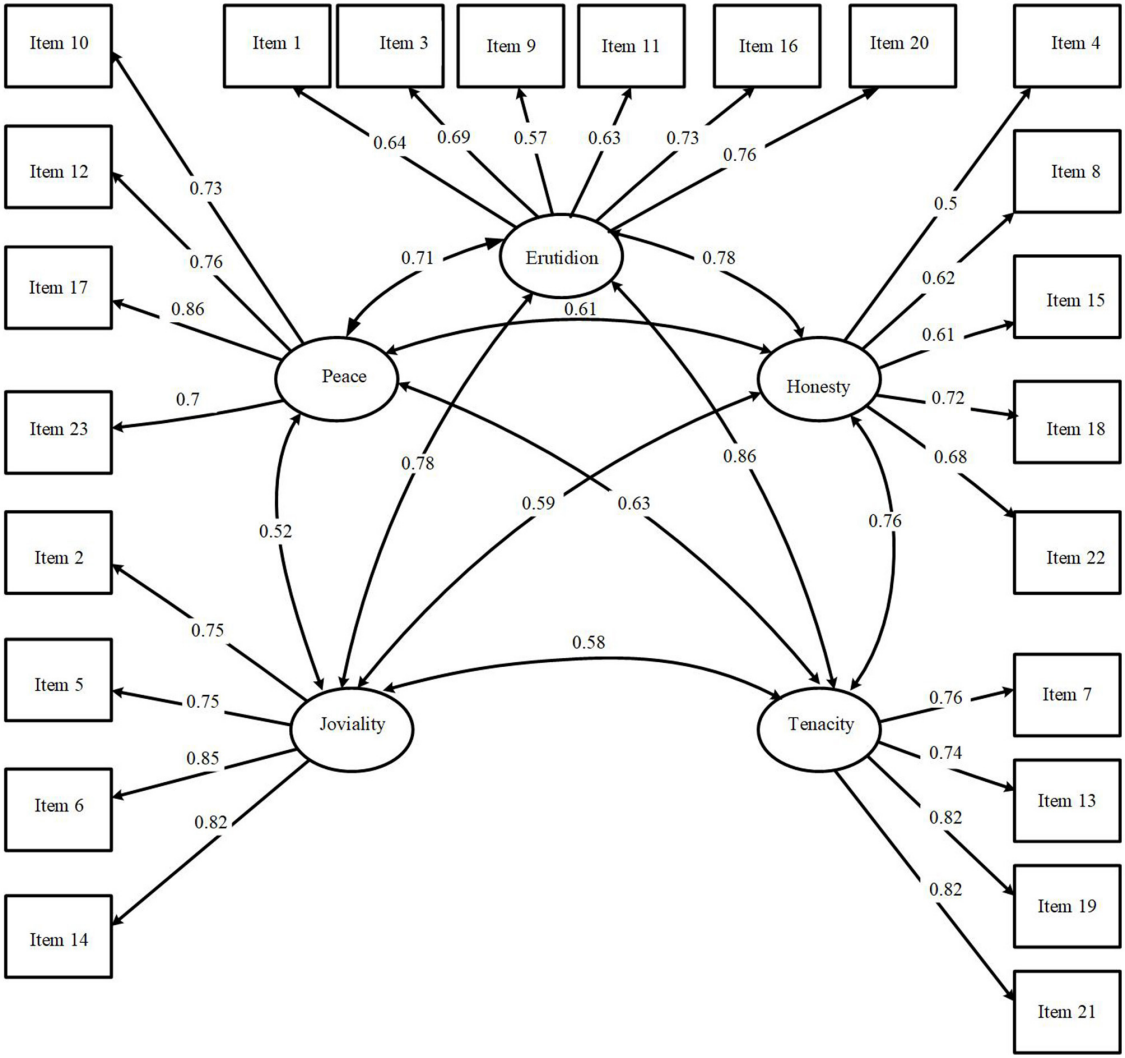
Diagram of the confirmatory factor analysis of the High Five Model Factors.

Values close to or greater than 0.95 were considered acceptable for CFI; values close to or less than 0.08 for SRMR; and values less than 0.07 for RMSEA, with the upper limit of its confidence interval below 0.08, as indicators of good model fit to the data. The model considers the inventory to be multidimensional, strictly adhering to the framework proposed by Cosentino and Castro Solano (2017). In addition, there is a closer alignment with the expected absolute, incremental, and parsimony indices for validating the construct, as shown in the diagram in Figure 1. The CFA results, using the diagonal weighted least squares (DWLS) estimator on a sample of 1, 007 adults, were as follows: CMIN or χ^2^(220) = 859.969, *p* < 0.001; robust CFI = 0.989, RMSEA = 0.054 (confidence interval between 0.050 and 0.058), and SRMR = 0.045. Therefore, the model demonstrated a good fit to the data. The graphical model is shown in Figure 1.

Shown in Table 3 the factor loadings obtained from the model. No coefficient were observed to fall below 0.500, with only item 4: “I have loyalty” from the honesty dimension having a value of 0.500. Overall, the results demonstrate a good saturation of items with their latent variables.

**Table 3:** Estimated and standardized factor loadings of observed variables for the HFI-E.

**Latent variable**		**Estimate**	**Std.Err**	**Z - value**	***P* (>|z|)**	**Std.lv**	**Std.all**
Erudition	Ítem 1	1.000				0.906	0.640
	Ítem 3	1.099	0.045	24.284	0.000	0.996	0.691
	Ítem 9	0.873	0.059	14.872	0.000	0.791	0.571
	Ítem 11	0.893	0.057	15.773	0.000	0.809	0.631
	Ítem 16	1.257	0.053	23.557	0.000	1.138	0.734
	Ítem 20	1.114	0.050	22.114	0.000	1.009	0.758
Peace	Ítem 10	1.000				1.084	0.727
	Ítem 12	1.024	0.063	16.142	0.000	1.110	0.755
	Ítem 17	1.148	0.061	18.762	0.000	1.244	0.857
	Ítem 23	1.045	0.052	20.255	0.000	1.133	0.703
Joviality	Ítem 2	1.000				1.184	0.754
	Ítem 5	0.937	0.047	19.911	0.000	1.109	0.747
	Ítem 6	1.094	0.039	28.404	0.000	1.295	0.852
	Ítem 14	1.075	0.038	27.990	0.000	1.272	0.816
Honesty	Ítem 4	1.000				0.478	0.500
	Ítem 8	1.334	0.139	9.620	0.000	0.638	0.617
	Ítem 15	0.981	0.120	8.187	0.000	0.469	0.613
	Ítem 18	2.045	0.213	9.599	0.000	0.977	0.724
	Ítem 22	1.568	0.156	10.048	0.000	0.749	0.684
Tenacity	Ítem 7	1.000				1.053	0.760
	Ítem 13	0.963	0.044	21.884	0.000	1.014	0.743
	Ítem 19	0.993	0.042	23.882	0.000	1.046	0.818
	Ítem 21	1.039	0.041	25.596	0.000	1.094	0.817

#### 6.1.2 Reliability of the HFI-E

The internal consistency analysis of the HFI-E was conducted using reliability coefficients α and ω. Table 4 indicates that overall reliability is generally high across nearly all indicators, with calculated coefficients ≥ 0.80. Only the honesty dimension exhibited acceptable consistency below 0.80. Evaluation of each item's contribution to the dimension revealed that removing any items did not enhance this level of reliability.

**Table 4:** Internal consistency analysis of the Ecuatorian version of the HFI-E.

**HFI-E factors**	**Cronbach's α**	**McDonald's ω**
Erudition	0.828	0.832
Peace	0.846	0.844
Joviality	0.870	0.871
Honesty	0.765	0.773
Tenacity	0.862	0.864

## 7 Discussion

The adaptation of the measurement instrument demonstrated adequate psychometric properties in terms of reliability and validity in its Ecuadorian version, applied using a CFA, where the model showed a good fit to the data with a robust *CFI* = 0.989. This result is consistent with the work of Cosentino and Castro Solano in 2017, who found a robust *CFI* = 0.963 in an adult population in Argentina, and also aligns with the study by Castro Solano and Cosentino (Solano and Cosentino, 2019), who reported a robust *CFI*>0.96 in university students.

Similarly, this study demonstrates internal consistency with α or ω≥0.80 for each factor or dimension, a finding that is consistent with the study by Cosentino and Castro Solano in 2017, which reported alpha and omega reliabilities above 0.80 for each factor in an adult population in Argentina. It also aligns with the work of Castro Solano and Cosentino (Solano and Cosentino, 2019), who found alpha and omega reliabilities above 0.80 for each factor.

These results confirm the proposed hypothesis as the demonstrated psychometric properties suggest that the HFI-E is recommended for assessing positive personality factors in the Ecuadorian population due to its reliability and validity.

## 8 Conclusion

Presented research addresses the study of positive personality traits through the FHI, providing a solid foundation for promoting personal development and psychological wellbeing. The application and validation of the psychometric properties of the HFI in the Ecuadorian context marks a significant milestone in this field of study. This advancement represents a substantial contribution to personality psychology and psychometric assessment, offering a valuable resource for investigating positive personality traits.

The reliability and validity demonstrated by the HFI in its Ecuadorian adaptation reinforce the need for culturally adapted psychometric instruments that allow for accurate and relevant assessment of personality traits within different contexts. Therefore, the incorporation of the HFI in Ecuador not only expands the possibilities for applying this instrument but also enriches the body of scientific literature related to personality structure and its various cultural expressions, thus fostering a more inclusive and diverse approach to psychological research.

## Data Availability

The original contributions presented in the study are included in the article/supplementary material, further inquiries can be directed to the corresponding author.
